# Amputation at the Knee-Joint

**Published:** 1868-06

**Authors:** 


### Amputation at the Knee-Joint.

Dr. Thomas M. Markoe, of New York, has succeeded in collecting records of 51 cases of amputations through the knee-joint performed in this country since 1856. In the year mentioned he published an account of 18 cases operated upon by American surgeons, with only 5 deaths. Of the 51 operations now mentioned in his paper to the *New York Medical Journal*, 39 were necessitated by accidents or injuries, and of these, 20, or more than 51 per cent. died; of the remaining 12, operated upon for conditions arising from disease in the leg or joint, but 2, or about 17 per cent. died. This is a very favorable showing, and would be still more so if 4 successful cases, the notes of which had not arrived in time for publication, were included. There would then be 55 cases, with 40 per cent. of deaths. And here let us revert to what appears to be an error; 22 of the 55 recent cases died, and according to Gross' Surgery, 5 deaths took place among the 18 reported in 1855. This would make 27 instead of 25 deaths in the total number, and would raise the per cent. from 34, as given by Dr. Markoe, to 37. Dr. Markoe looks very favorably upon the disarticulation as compared with amputation through the thigh, but we conceive that he makes a serious error in taking into his calculations all cases of amputation of the thigh, whether of the upper, middle or lower thirds. Every one knows how much more fatal operations are as they approach the trunk; indeed, Dr. Markoe claims this as a reason why disarticulation should be preferred, yet he fails to exclude all but amputations through the lower third, to which alone, it is manifest, disarticulations should be compared. Again, his comparisons, it strikes us, would be more valuable if made with reference to whether disease or accident necessitated the amputation. A preponderance of either of these classes would greatly modify the result, as may be seen at a glance, by turning to the figures quoted above. The points made by Dr. Markoe are: 1st, That the ancient prejudice against this operation and the objections to it on the ground of the danger of opening so large a joint as that of the knee, are unfounded, at least, in the degree to which they are practically important. 2d, There seems good reason to believe that the shock to the system, and the demand upon its reparative power, are less in amputation at the knee-joint, than in amputation higher up. 3d, The condition of the stump during the progress of cure, is more favorable and less distressing to the patient because the muscles not having been divided, voluntary motion is not prevented nor is it painful, and retraction, by which the end of the bone may be uncovered, cannot take place. 4th, The bone being unwounded, the dangerous and troublesome accidents likely to follow exposure of the medulla by the saw, such as necrosis, osteo-myelitis and pyemia, are avoided. 5th, The stump remaining after knee-joint amputation, is much more serviceable than those following operations higher up, permitting the whole weight of the body to be borne upon it with ease and comfort.—*Pacific Medical Journal.*

